# Exploring the importance of controlling heteroskedasticity and heterogeneity in health valuation: a case study on Dutch EQ-5D-5L

**DOI:** 10.1186/s12955-022-01989-9

**Published:** 2022-05-25

**Authors:** Suzana Karim, Benjamin M. Craig, Catharina G. M. Groothuis-Oudshoorn

**Affiliations:** 1 University of South Florida, 4202 E Fowler Ave, Tampa, FL 33620 USA; 2 University of Twente, Enschede, The Netherlands

**Keywords:** Health valuation, Best–worst scaling, Heteroskedasticity, Scale heterogeneity, EQ-5D

## Abstract

**Background:**

Respondents in a health valuation study may have different sources of error (i.e., heteroskedasticity), tastes (differences in the relative effects of each attribute level), and scales (differences in the absolute effects of all attributes). Although prior studies have compared values by preference-elicitation tasks (e.g., paired comparison [PC] and best–worst scaling case 2 [BWS]), no study has yet controlled for heteroskedasticity and heterogeneity (taste and scale) simultaneously in health valuation.

**Methods:**

Preferences on EQ-5D-5L profiles were elicited from a random sample of 380 adults from the general population of the Netherlands, using 24 PC and 25 BWS case 2 tasks. To control for heteroskedasticity and heterogeneity (taste and scale) simultaneously, we estimated Dutch EQ-5D-5L values using conditional, heteroskedastic, and scale-adjusted latent class (SALC) logit models by maximum likelihood.

**Results:**

After controlling for heteroskedasticity, the PC and BWS values were highly correlated (Pearson's correlation: 0.9167, CI: 0.9109–0.9222) and largely agreed (Lin's concordance: 0.7658, CI: 0.7542–0.7769) on a pits scale. In terms of preference heterogeneity, some respondents (mostly young men) failed to account for any of the EQ-5D-5L attributes (i.e., garbage class), and others had a lower scale (59%; p-value: 0.123). Overall, the SALC model produced a consistent Dutch EQ-5D-5L value set on a pits scale, like the original study (Pearson's correlation:0.7295; Lin's concordance: 0.6904).

**Conclusions:**

This paper shows the merits of simultaneously controlling for heteroskedasticity and heterogeneity in health valuation. In this case, the SALC model dispensed with a garbage class automatically and adjusted the scale for those who failed the PC dominant task. Future analysis may include more behavioral variables to better control heteroskedasticity and heterogeneity in health valuation.

**Highlights:**

- The Dutch EQ-5D-5L values based on paired comparison [PC] and best-worst scaling [BWS] responses were highly correlated and largely agreed after controlling for heteroskedasticity.
- Controlling for taste and scale heterogeneity simultaneously enhanced the Dutch EQ-5D-5Lvalues by automatically dispensing with a garbage class and adjusting the scale for those who failed the dominant task.
- After controlling for heteroskedasticity and heterogeneity, this study produced Dutch EQ-5D-5L values on a pits scale moderately concordant with the original values.

## Introduction

Developed by the EuroQol group in 2005, the EQ-5D-5L instrument provides a widely used descriptive system for health valuation in multiple languages [1]. This descriptive system expresses a person's health along with five attributes, i.e., mobility, self-care, usual activities, pain/discomfort, and anxiety/depression. Each attribute has five levels (no problems, slight problems, moderate problems, severe problems, and unable to/ extreme problems) describing the severity of the person's health problems. Using this system of five five-level attributes, health valuation studies may ask respondents about their preferences regarding its 3125 possible health profiles (5^5^).

In general, collecting ordinal responses using choice tasks, such as PC and BWS, is gaining widespread use in health economics and policy [2, 3]. Methodological advances in health preference research (HPR) have been applied successfully in eliciting patient and community preferences for a wide range of health care interventions [4]. Many literature reviews have been conducted that show the gaining interest in HPR [2, 5]. As a potential methodological extension, some researchers proposed including more choice tasks, such as ranking and best–worst scaling (BWS), as complements or alternatives to the time trade-off (TTO) tasks in the EQ-VT protocol [6–9]. Furthermore, many believe that choice tasks with their ordinal responses were less cognitively burdensome than cardinal tasks with their indifference responses [10–12]. The EQ-VT protocol currently includes some PC as a complement to the TTO to better understand preferences between EQ-5D-5L profiles; therefore, there seems to be an opportunity to include additional choice tasks within the protocol. In this project, we conducted a Dutch EQ-5D-5L valuation study, including PC and BWS tasks, to explore a natural extension to the EQ-VT protocol. The valuation is done in a pits scale rather than the conventional QALY due to lack of the life span attribute [13]. We proposed this project in hopes that BWS might serve as a possible alternative or addition for PC tasks in the EQ-VT protocol. Specifically, the single-profile (or case 2) task is one of the three BWS tasks [14]. Unlike a PC, where respondents choose between two EQ-5D-5L profiles, respondents in a case-2 BWS task face a single EQ-5D-5L profile (like a TTO task), making this task more coherent with the TTO task. In the case-2 BWS task, the respondent indicates the best and the worst attribute levels within the given profile. In this study, we hypothesize that the EQ-5D-5L values estimated using the PC and BWS responses agree.

### Heteroskedasticity and heterogeneity in health valuation

Heteroskedasticity and heterogeneity have been identified as key limitations to the analysis and interpretation of preference evidence, particularly ordinal responses[15]. A recent review on heterogeneity analyses in HPR showed that most published studies analyzed heterogeneity without controlling for heteroskedasticity or differential scaling [3]. This paper further contributes to HPR by demonstrating the implications of controlling heteroskedasticity and heterogeneity in health valuation as well as separating taste and scale heterogeneity.

Like other observable differences [16], heteroskedasticity refers to differences in variance by observable factors, such as task-level or individual-level factors. In a heteroskedastic logit, its variance may vary between tasks systematically in response to task complexity and the number of choice alternatives, attribute differences, or individual behavioral differences [15, 17]. In this study, we hypothesize that variance varies by task sequence and task type and that controlling this heteroskedasticity can reduce uncertainty in EQ-5D-5L values. Heteroskedasticity is not a form of preference heterogeneity because the difference in variance is derived from a difference in behavior (e.g., task sequence), not preference.

Apart from heteroskedasticity, we also examine two types of preference heterogeneity [18]. First, groups of respondents like or dislike different alternatives in a systematic way that reflects the relative importance of the attributes (i.e., taste classes). Taste heterogeneity refers to differences in the relative effects of each attribute level. For example, some respondents may place a greater weight on functioning and others on feeling (e.g., pain/discomfort, anxiety/depression). Alternatively, there can be a group of respondents who fail to account for any of the EQ-5D-5L attributes, and by summing up their preference information creates coefficient estimates of garbage class. The responses of people who belong to a garbage class may show the probability of choosing the best (11111) over the worst (55555) EQ-5D-5L profile is near 50%. Second, groups may like or dislike alternatives systematically that reflect the absolute value of all attributes (i.e., scale classes). Scale heterogeneity refers to more subtle differences in the absolute effects of all attributes (compared to garbage classes), and scale classes may be related to the respondents' difficulty distinguishing between alternatives (e.g., more indifference with a lower scale value).

Estimating differences in attribute importance between respondents without controlling for scale heterogeneity can often mislead the interpretation of taste heterogeneity, which is confounded by scale heterogeneity [19]. Using the information on the respondents, a scale-adjusted latent class (SALC) model [20] can disentangle taste and scale heterogeneity simultaneously by identifying latent classes of persons who differ in their relative importance (taste classes), as well as latent scale classes—groups of people who differ by how intense (or indifferent) their preferences are. In this paper, heteroskedasticity is associated with observable differences in scale between tasks (e.g., task sequence), and scale heterogeneity is associated with latent differences in scale between individuals (e.g., failing the PC dominance task). The SALC model allows for heteroskedasticity and two forms of heterogeneity, and we hypothesize that controlling for all three can enhance health valuation. Given this background, this study is aimed to run a case analysis on a Dutch EQ-5D-5L valuation dataset with the following objectives. First, we examined the effects of controlling heteroskedasticity by comparing the results of the conditional and heteroskedastic logit. Second, we illustrated the EQ-5D-5L values based on the PC and case-2 BWS responses and assessed their correlation and agreement. Third, we estimated EQ-5D-5L values using the scale-adjusted latent class (SALC) logit models, which control for taste and scale heterogeneity as well as heteroskedasticity. Finally, we compare the Dutch EQ-5D-5L values to the original values produced using the EQ-VT protocol [21].

## Methods

### Overview

In September 2016, Dutch respondents were recruited from a marketing panel (Survey Sampling International) to complete computer-based interviews via an online survey instrument. We did not aim for a fully representative sample but sampled from groups with known EQ-5D-5L impairments. We aimed to sample 300 subjects stratified by domain and severity of health problems captured by the EQ-5D-5L. To facilitate the analysis of preference heterogeneity, all respondents completed the same PC and case-2 BWS tasks using the same series of EQ-5D-5L profiles. Examples of the PC and BWS tasks can be found in Appendix 1.

### EQ-5D-5L profiles

Using the EQ-5D-5L descriptive system, the five five-level attributes can be described by a 5-digit vector of the attribute levels, where the position of the integer refers to the attribute, while the integer itself refers to the attribute level. For example, EQ-5D-5L profile '32512' would describe moderate problems walking about, some problems washing or dressing self, unable to perform usual activities, no pain or discomfort, and some anxiety or depression.

### Experimental design

The BWS' Health profile A' design is based on an orthogonal main effects plan (OMEP) [22] that, in the case of the EQ-5D-5L, consists of 25 profiles. With these 25 profiles, in principle, it is possible to estimate 24 individual BWS level parameters. By *rotating the OMEP coding*,^1^ a design was obtained with the minimal number of only one attribute at an extreme level, resulting in 15 out of 25 best choices with at least two attributes with the same lowest level and 16 out of 25 worst choices with at least two attributes with the same highest level. Therefore, at least 31 − 24 = 7 degrees of freedom to estimate a model for every respondent in case the other 19 choices were non-informative. The chosen health profiles are listed in Appendix 1 (Table 5). Moreover, the final design contained no states with all attributes at the same level, which would make the task excessively difficult, and the PC contained only one dominant comparison out of the 25 comparisons. Overall, it is not a representative sample, but more a stratified sample to the severeness of disease. The questionnaire was designed in a fashion that respondents first were asked to perform the BWS case 2 task with profile A.

Next, for the PC task, the 'Health profile B' that was added as a comparator to the BWS' Health profile A' was always the same profile, namely (24242), a state close to the center of the health-profile continuum (based on Devlin et al. [23]) that has three attributes at the same level, and the other two as well. Such a constant comparator design reduces efficiency to around 40–50% but provides the only currently known compromise possible between the needs of the case 2 BWS and the needs of the PC tasks [24]. This particular dual design appears unusual but is important in that it has properties that reflect the BWS case 2/PC relationship (investigation of "how I rescale my BWS case 2 estimates into PC-space") and practical benefits (minimizing cognitive load in the PC by familiarizing the respondent with profile A, then adding a constant, known, state B). A sample question of both types of tasks can be found in Appendix 1, Fig. 4.

### Analysis

The final analysis dropped the dominant task from the PC question. Descriptive statistics were used to summarize respondents' characteristics and response feasibility of PC and BWS tasks. To maximize the use of the available data, we implemented a hybrid modeling approach that incorporated all PC and BWS responses to produce the Dutch EQ-5D-5L value set. Conditional logit model, heteroskedastic conditional logit, and heteroskedastic scale-adjusted latent class (SALC) model were estimated by maximum likelihood to illustrate the values for all 3125 EQ-5D-5L profiles [25]. The main effects of each model are shown as incremental changes in the level of severity on a pits scale where value (55555) = 0 and value (11111) = 1 [13]. Unlike EQ-VT studies, the study did not use the TTO or include any preferences evidence on "dying immediately;" therefore, the main effects cannot be reported on a quality-adjusted life-year (QALY) scale. Statistical analyses were done in R 4.0.2 [26–28]. A significance level of 0.05 was considered statistically significant.

#### Main-effect specification of EQ-5D-5L Values

To aid the interpretation of the BWS responses, we envisioned a profile of '00000' that represents a hypothetical ideal. The BWS specification includes twenty incremental variables, each representing the loss in health values for increasing severity from one level to the next of the same dimension, as well as five ancillary variables associated with a change in level from zero to one, which is outside the EQ-5D-5L descriptive system. The primary difference between the best and the worst responses is that the sign of the incremental variables switches (i.e., for best, the incremental variable is negative; for worst, the variable is positive). The hypothetical ideal is not relevant for the interpretation of the PC responses; therefore, its specification includes only the twenty incremental variables.

The twenty main-effect coefficients describe the value of the EQ-5D-5L profiles on a pits scale. The coefficients of the five ancillary variables have no effect on the EQ-5D-5L values; therefore, these estimated coefficients are reported in Appendix 1. Due to the identification problems of case-2 BWS, only four of the five ancillary parameters can be non-zero; therefore, we constrained the smallest ancillary parameter to zero, which has no effect on the EQ-5D-5L values.

#### Heteroskedasticity and differences by task

Overall, each PC and BWS response is a multinomial choice (from two and five alternatives, respectively) that reflects a respondent's preferences taking into account the 20 and 25 incremental variables, respectively. The conditional logit model assumes homogeneous preference and independent and identically distributed (IID) errors. Relaxing the IID assumption introduces the heteroskedastic conditional logit (HCL) model [29], where the scale parameter (inversely related to the variance) is an exponential function of observable factors that identify the source of differential variance and constrains the scale to be non-negative. The differential variance may be associated with individual level, choice set/task level, or alternative level characteristic variables. To avoid confounding between heteroskedasticity and scale heterogeneity, the scale parameter in this paper depends on only task-level variables, namely task sequence and task type (e.g., best/worst/paired comparison).

Furthermore, we estimated the heteroskedastic logit by task (i.e., BWS case 2 and PC) characteristics, computed the PC and BWS values using the interaction results, and assessed their correlation and agreement (Pearson's correlation and Lin's concordance, respectively).

#### Heterogeneity and EQ-5D-5L Values

The SALC model (model formulation in Appendix 2) allows for preference heterogeneity through latent classes. Taste classes represent groups that share the relative effects of each attribute level, and scale classes represent groups that share the absolute effects of all attributes. The likelihood that each individual belongs to a specific group is known as a respondent's grade-of-membership (GOM) and may be associated with their observable characteristics. In the analysis, we hypothesize that individuals' demographics, socio-economic variables, and health conditions are associated with taste class membership. The scale class, which identifies the irregularities and idiosyncratic features of choice behavior that are not particularly associated with any attribute level, rather captures the variability across subjects, tasks, and objects are identified by individual's age, education level, gender, competency level (whether passed the dominant task), and perception on the difficulty level between the two question types.

As an extension of the HCL [30, 31], the standard SALC model [20] identifies differences in scale by latent groups (i.e., scale classes), but scale remains constant within each scale class. [18, 32]. A SALC model can allow heteroskedasticity by letting the scale factor vary by observable factors within each scale class (i.e., heteroskedastic SALC).

As the number of classes both for the scale and taste classes is decided prior to the analysis rather than identified from estimation, a series of classes is usually estimated, and the best-fitted model is based on statistical and substantive criteria (i.e., BIC, AIC, CAIC) [33]. However, in empirical analysis, factors like a smaller size, complexity in the model, and low efficiency may cause identification problems for a higher dimension solution with many latent classes. This study only collected 380 respondents; therefore, the SALC model includes only two taste and two-scale classes.

In order to compare these values with the original Dutch EQ-5D-5L values [21], the original values were transformed to a pits scale, and their relationship was illustrated using a scatter plot and estimates using Pearson's correlation and Lin's concordance.

## Results

### Demographics

After excluding the dominant pair from the PC task, the analysis included 24 PC tasks and 25 BWS tasks. In total, 385 respondents completed the questionnaires, from which five were excluded due to engaging in click-through on the PC (no variation in their responses), so subsequent analyses are based on the remaining 380 respondents. Fifty-two percent (n = 198) of the respondents were male (Table 1). Respondents were almost equally divided among the age group 16 to 35, 36 to 55, and above 55. More than half of the respondents had a middle education (n = 197) compared to thirty-five percent (n = 131) having high education. Fifty-seven percent (n = 217) reported having a chronic illness.

**Table 1:** Descriptive statistics sample (n = 380)

Characteristic		n (%)
Gender (N, %)	Men	198 (52.1%)
	Woman	182 (47.9%)
Age	16- 35	124 (32.6%)
	16 – 55	117 (30.8%)
	55 above	149 (36.6%)
Educational level	Low	52 (13.7%)
	Middle	197 (51.8%)
	High	131 (34.5%)
Chronical Illness	Yes	217 (57.1%)
	No	163 (42.9%)
VAS score Health	< 70	200 (52.6%)
	70 above	180 (47.4%)
Difficulty BWS	Easy	71 (18.7%)
	Not easy / not difficult	192 (50.5%)
	Difficult	117 (30.8%)
Difficulty PC	Easy	61 (16.1%)
	Not easy / not difficult	173 (45.5%)
	Difficult	146 (38.4%)
Easiness BWS/PC	BWS	135 (35.5%)
	No preference	173 (45.5%)
	PC	72 (19.0%)
Failed dominant task in PC		72(19.0%)

### Feasibility

Thirty-one percent (n = 117) found the best–worst questions difficult, compared to thirty-eight percent (n = 146) for the PCs (Table 1). Seventy-two out of 380 respondents preferred in terms of difficulty the PC questions over the best–worst questions. Almost half of the respondents (n = 173) had no preference. Remarkably, from those indicating BWS easier than PC rated 9/380 = 2.3% the difficulty of PC lower (less difficult) than BWS; those indicating PC is easier than BWS rated 13/380 = 3.4% the difficulty of BWS lower than PC. And finally, from those indicating no preference in the easiness of BWS or PC gave 42/380 = 11.1% a different level of difficulty to the two methods. Also, 72 of the total respondents failed the dominant task.

### Difference between homoskedastic and heteroskedastic results

Table 2 showed the main effect estimates of the conditional logit (CL) model and heteroskedastic conditional logit (HCL) model. The HCL model fitted better by lowering the BIC value by 1666.29 (CL BIC: 64,458.32, and HCL BIC: 62,792.03). The correlation between the 3125 values measured by the CL and HCL estimates showed a high correlation (Pearson's correlation coefficient is 0.9953 (CI: 0.9950–0.9956) and Lin's concordance correlation coefficient 0.9927 (CI: 0.9922–0.9932)) (Appendix 1 Fig. 5). In both models, one incremental coefficient is negative (i.e., the change in severity from severe to extreme under usual activity) but insignificant (CL coefficient: − 0.0003 p-value: 0.956; HCL coefficient: − 0.0011, p-value:0.878). The sequence of completing tasks has a positive effect on the scale parameter (0.8419; p < 0.001), and its square has a negative effect ( − 0.7427, p < 0.001), indicating that scale increased (i.e., less random responses) up to fourteen tasks and decreased after that (Fig. 2) with overall p-value < 0.001. Also, the effect of the PC task on the scale parameter is significantly negative ( − 0.9930, p-value < 0.001), and the effect of the best task is significantly positive (0.2424, p-value < 0.001) (Appendix 1 Table 6). Controlling heteroskedasticity had little effect on the standard errors; the standard error decreased in 8 of the 20 estimated parameters (Appendix 1 Table 6).

**Table 2:** Conditional, heteroskedastic, and interaction model (controlling heteroskedasticity)

					Interaction				
	Conditional		Heteroskedastic		Paired comparison		Best worst scaling		
	Coef	p-value	Coef	p-value	Coef	p-value	Coef	p-value	p-value*
Mobility									
Level 1–2	0.0879	< 0.001	0.0726	< 0.001	0.0874	< 0.001	0.0261	0.192	0.001
Level 2–3	0.0331	< 0.001	0.0340	< 0.001	0.0232	< 0.001	0.0478	0.002	0.917
Level 3–4	0.1073	< 0.001	0.1097	< 0.001	0.1103	< 0.001	0.1078	< 0.001	0.451
Level 4–5	0.0134	0.015	0.0059	0.398	0.0025	0.662	0.0308	0.091	0.931
Self-care									
Level 1–2	0.0634	< 0.001	0.0623	< 0.001	0.0652	< 0.001	0.0803	< 0.001	0.278
Level 2–3	0.0062	0.270	0.0271	< 0.001	0.0276	< 0.001	0.0545	0.028	0.273
Level 3–4	0.0572	< 0.001	0.0370	< 0.001	0.0498	< 0.001	0.0123	0.602	0.599
Level 4–5	0.0170	< 0.001	0.0164	0.003	− 0.0002	0.974	0.0559	0.001	0.999
Usual activity									
Level 1–2	0.0430	< 0.001	0.0588	< 0.001	0.0685	< 0.001	0.0648	0.005	0.968
Level 2–3	0.0299	< 0.001	0.0280	< 0.001	0.0243	< 0.001	− 0.0784	0.001	0.611
Level 3–4	0.0987	< 0.001	0.1002	< 0.001	0.1008	< 0.001	0.1129	< 0.001	< 0.001
Level 4–5	− 0.0003	0.956	− 0.0011	0.878	− 0.0046	0.423	0.0312	0.083	0.999
Pain/discomfort									
Level 1–2	0.0682	< 0.001	0.0802	< 0.001	0.0854	< 0.001	0.0061	0.728	0.739
Level 2–3	0.0125	0.025	0.0324	< 0.001	0.0325	< 0.001	0.0637	0.010	0.194
Level 3–4	0.1244	< 0.001	0.1034	< 0.001	0.1160	< 0.001	0.0856	< 0.001	0.004
Level 4–5	0.0297	< 0.001	0.0336	< 0.001	0.0139	0.015	0.0970	< 0.001	0.999
Anxiety/depression									
Level 1–2	0.0649	< 0.001	0.0605	< 0.001	0.0738	< 0.001	0.0106	0.636	0.002
Level 2–3	0.0486	< 0.001	0.0444	< 0.001	0.0319	< 0.001	0.0568	< 0.001	0.922
Level 3–4	0.0565	< 0.001	0.0634	< 0.001	0.0623	< 0.001	0.0735	< 0.001	0.710
Level 4–5	0.0384	< 0.001	0.0310	0.003	0.0296	< 0.001	0.0575	0.001	0.926

*p-value showed the significant difference between the PC and BWS coefficient within the heteroskedastic logit

Coefficients are showing as incremental change in the level of severity on a pits scale where value (55555) = 0 and value (11111) = 1; Detailed results are in Appendix 1

### Differences between the PC and BWS results

**Fig. 1:**
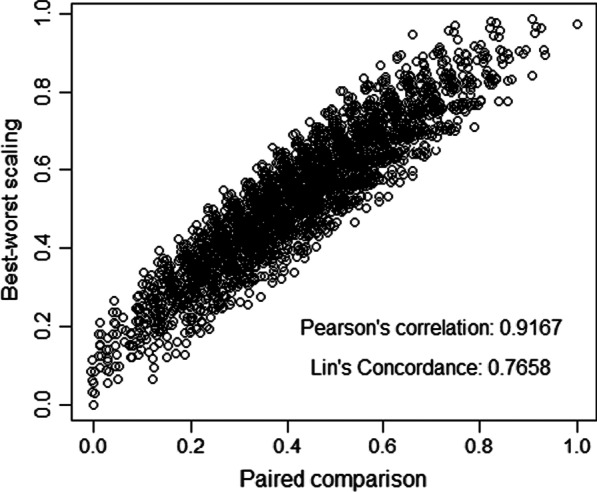
Scatter plot of 3125 EQ-5D-5L profiles for heteroskedastic model. *values were estimated in a pits scale where v (55555) = 0 and v (11111) = 1. 95% Confidence interval for Pearson’s correlation 0.9109–0.9222, and for Lin’s concordance: 0.7542–0.7769

Table 2 also showed the main-effect coefficients of PC and BWS for the heteroskedastic logit model. In the PC estimates, 17 out of 20 coefficients were significant (p < 0.05); however, two coefficients were negative but insignificantly different from zero. Under BWS, 13 coefficients were significant, with one significant negative estimate. Only four coefficients have shown a significant difference by task, and the largest difference is 0.1027. Converting the 3125 EQ-5D-5L values into a pits scale, we measured the correlation between PC and BWS values. (Fig. 1). Between the two 3125 EQ-5D-5L profiles, Pearson's correlation coefficient is 0.9167 (CI: 0.9109–0.9222), and Lin's concordance correlation coefficient is 0.7658 (CI: 0.7542–0.7769). The median absolute difference in the difference between PC and BWS values has 0.0732 (interquartile range 0.0592 to 0.1565).

### Taste and scale heterogeneity

The SALC model increased model fit compared to homogeneous models by achieving the lowest BIC value (56,698.35). Table 3 showed the main-effect coefficients of the two taste classes. Taste class 1 had consistent parameters with non-negative values, and 19 of them were significant (p < 0.050). In all the attributes, changing levels from moderate to severe problems led to the greatest reduction in value. Based on this evidence, taste class 1 is referred to as a Dutch EQ-5D-5L value set on the pits scale.

**Table 3:** Two taste classes of the scale-adjusted latent class (SALC) model

	Taste class 1 EQ-5D-5L values set		Taste class 2 garbage class	
	Coef	p-value	Coef	p-value
Mobility				
Level 1–2	0.0586	< 0.001	0.2954	0.004
Level 2–3	0.0290	< 0.001	− 0.1132	0.348
Level 3–4	0.1205	< 0.001	0.1903	0.159
Level 4–5	0.0090	0.017	− 0.0825	0.518
Self-care				
Level 1–2	0.0553	< 0.001	− 0.2140	0.143
Level 2–3	0.0246	< 0.001	0.2202	0.127
Level 3–4	0.0630	< 0.001	− 0.0745	0.531
Level 4–5	0.0082	0.030	0.2378	0.048
Usual activity				
Level 1–2	0.0516	< 0.001	0.0386	0.794
Level 2–3	0.0297	< 0.001	− 0.0073	0.964
Level 3–4	0.1115	< 0.001	− 0.0431	0.767
Level 4–5	0.0002	0.967	0.0544	0.745
Pain/discomfort				
Level 1–2	0.0686	< 0.001	0.1218	0.438
Level 2–3	0.0290	< 0.001	− 0.0898	0.585
Level 3–4	0.1085	< 0.001	0.0930	0.575
Level 4–5	0.0346	< 0.001	− 0.1657	0.341
Anxiety/depression				
Level 1–2	0.0558	< 0.001	0.3042	0.037
Level 2–3	0.0412	< 0.001	0.0648	0.643
Level 3–4	0.0753	< 0.001	0.0241	0.885
Level 4–5	0.0262	< 0.001	0.1455	0.354
Prob (11111 > 55555) **	.998		.554	

Coefficients are showing as incremental change in the level of severity on a pits scale where value (55555) = 0 and value (11111) = 1; Detailed results are in Appendix 1

**The probability of choosing the best over the worst EQ-5D-5L profile is less than 56% in taste class 2 (calculating probability from the difference between v (11111) and v (55555) on a log-odds scale which is the pits value .2161; log (p/((1-p)) = 0.2161). In this study, taste class 2 is called the garbage class because the responses were unrelated to the ordinal attributes

On the other hand, taste class 2 had few significant parameters and eight inconsistent estimates. In this class, the probability of choosing the best over the worst EQ-5D-5L profile is 0.554 (Table 3), which is much smaller than the near-unanimous probability found in taste class 1 (0.998). Based on this evidence, taste class 2 is referred to as a garbage class.

Around 71% of the individuals belonged to taste class 1 and 29% in taste class 2 (Table 4). Looking at the grade-of-membership results, respondents in the garbage class are less likely to be female (odds ratio: 0.5173 95% CI: 0.3685 to 0.6661) and more likely to be younger (odds ratio: 2.4143; 95% CI: 1.6509 to 3.1777).

**Table 4:** Grade-of-membership (GOM) of the scale-adjusted latent class (SALC)

	GOM for taste class 2 garbage class (29% of respondents)		GOM for scale class 2 more random class (59% of respondents)	
	Coef	p-value	Coef	p-value
Intercept	0.5022	0.098	0.5453	0.117
Female	0.5173	0.022	1.0399	0.882
Age in years		< 0.001		0.875
16–35	2.4143	0.005	1.1669	0.554
36–54				
above 55	0.2100	0.011	1.4360	0.141
Educational attainment*		0.686		0.697
Low	0.6366	0.388	0.8678	0.705
Medium				
High	0.9140	0.839	0.9635	0.911
Chronic Illness				
Yes	1.0643	0.895		
VAS score Health				
Below 70	1.8346	0.249		
70 >	–			
Difficulty level				
Failed dominant task			3.1286	0.044
Found tasks easy			1.1337	0.834
Found tasks hard			0.6152	0.140

Results are shown on the odds ratio scale. For education, the lowest group included up to the primary, the medium group included secondary to associates, and the highest group included bachelor's degrees and above. The standard errors are shown in Appendix 1 Table 8

**Fig. 2:**
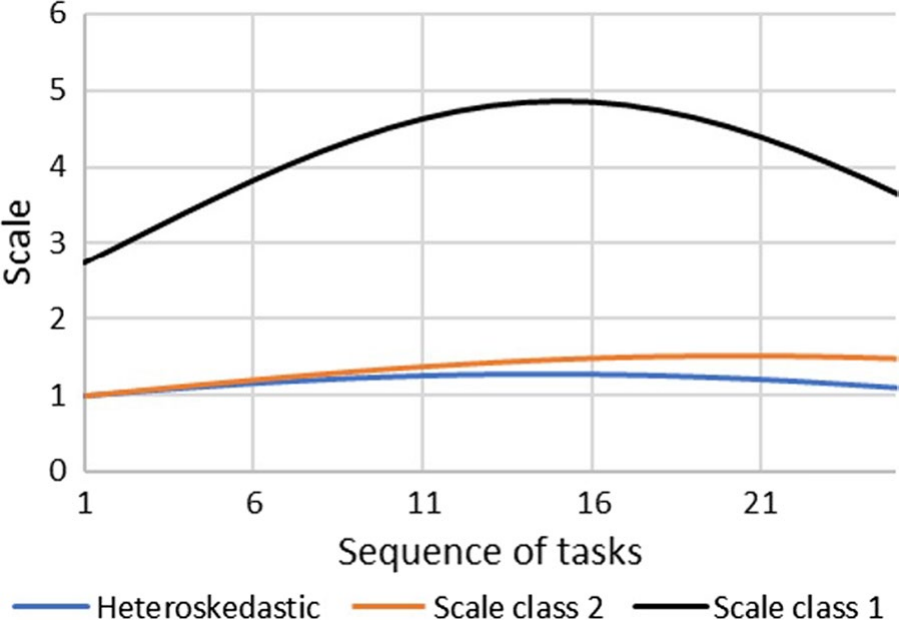
Heteroskedasticity: scale by the task sequence. *Scale coefficients were transformed into the original scale

The scale is lower in scale class 2 than in scale class 1, which implies scale class 2 has a higher variance (Appendix 1 Table 7). In scale class 1 (less random class), the effect of the sequence of tasks on the scale has a similar pattern as in the heteroskedastic model (Fig. 2). However, the coefficient of the sequence square was not significant (Appendix 1 Table 7 and Fig. 2). The effect of task type (i.e., PC or BWS) is the same across both classes, where PC is negatively associated with scale (i.e., increased uncertainty/randomness) and the best task under BWS is positively associated with scale factor (i.e., reduce uncertainty/randomness) (Appendix 1 Table 7). However, the coefficients were only significant in scale class 1. Around 41% of the respondents belong to scale class 1 and 59% to scale class 2. Respondents in scale class 2 were more likely to fail the PC dominant task (Table 4).

### Differences between the original and new Dutch EQ-5D-5L values

**Fig. 3:**
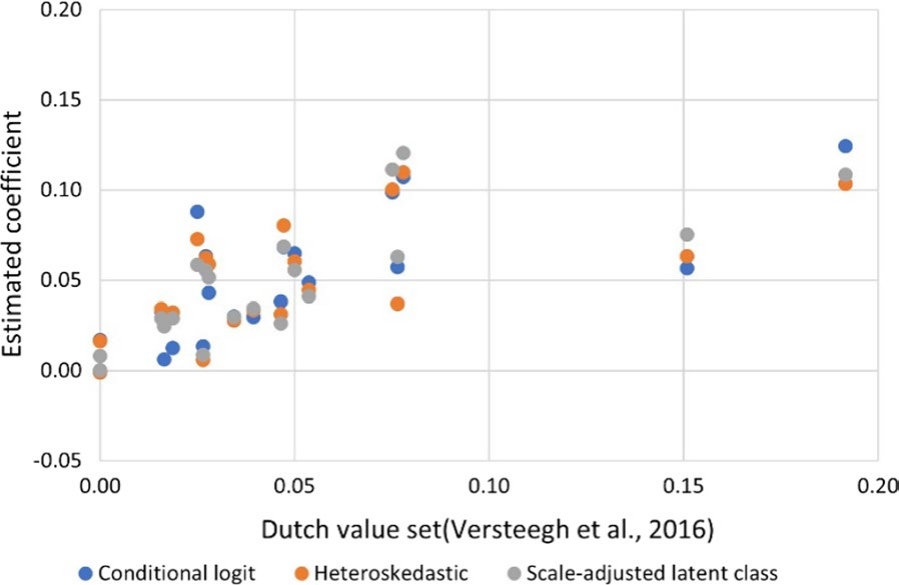
Comparing estimated coefficients with the Dutch value set. *Pearson's correlation coefficient for the 20 the conditional (0.6937), heteroskedastic (0.6321), and SALC (0.7295) coefficients

**Fig. 4:**
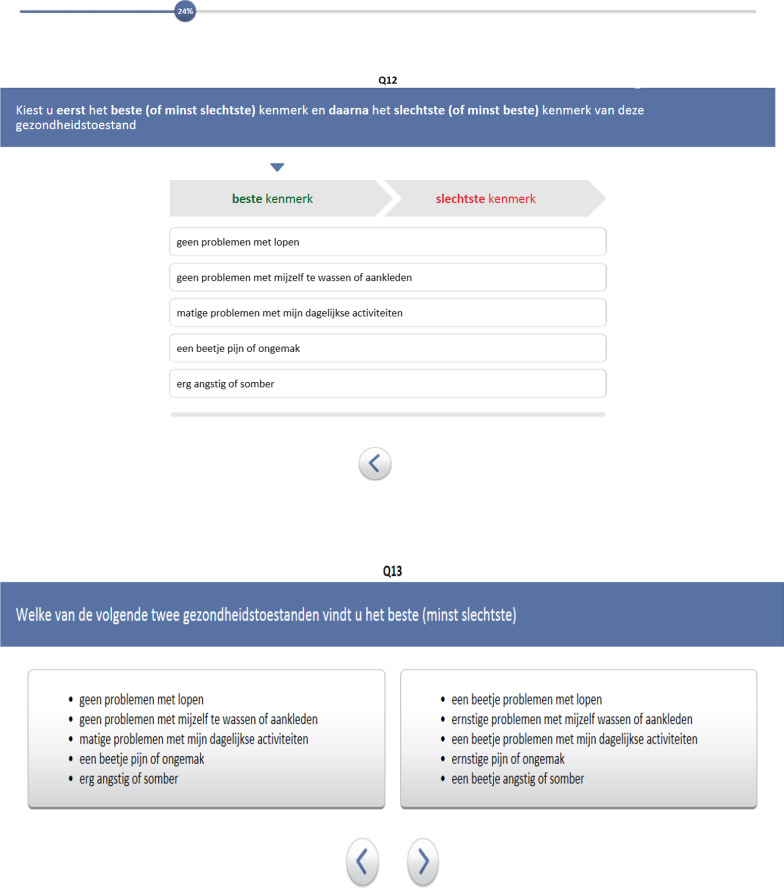
Sample Questions (in Dutch)

**Fig. 5:**
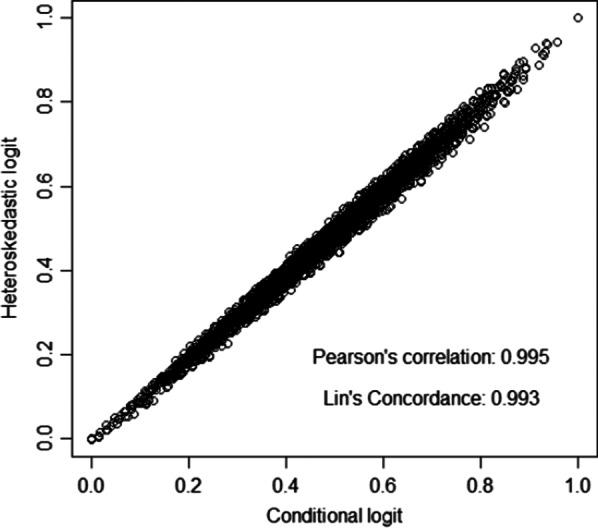
Scatter plot of 3125 EQ-5D-5L profiles for conditional logit and heteroskedastic model. *values were estimated in a pits scale where v (55555) = 0 and v (11111) = 1. 95% Confidence interval for Pearson’s correlation 0.9950–0.9956, and for Lin’s concordance: 0.9922–0.9932

Comparing the twenty main-effect coefficients estimated in this study with those of the original Dutch value set [21], the SALC coefficients had the highest correlation and agreement (Pearson’s correlation: 0.7295, CI: 0.4238–0.8860; Lin’s concordance: 0.6904, CI: 0.4098–0.8516), followed by conditional logit (Pearson’s correlation: 0.6937, CI: 0.3626–0.8693; Lin’s concordance: 0.6601, CI: 0.3554–0.8380) and heteroskedastic conditional logit (Pearson’s correlation: 0.6321, CI: 0.2632–0.8398; Lin’s concordance: 0.5817, CI: 0.2543–0.7894) (Fig. 3) [34]. Looking across the 3125 EQ-5D-5L values, the SALC values had the highest correlation and agreement (Pearson’s correlation: 0.9293, CI: 0.9244–0.9339; Lin’s concordance: 0.8835 CI: 0.8763–0.8903), followed by conditional (Pearson’s correlation: 0.9254, CI: 0.9203–0.9304; Lin’s concordance: 0.8689, CI: 0.8610–0.8764) and, heteroskedastic (Pearson’s correlation: 0.9226, CI: 0.9172–0.9277; Lin’s concordance: 0.8453, CI: 0.8364–0.8537).

## Discussion

Using a population-based sample from the Netherlands, we estimated the value of EQ-5D-5L profiles by task and controlling for heteroskedasticity and heterogeneity. Apart from heteroskedasticity, identifying taste heterogeneity often becomes difficult because of its confounding nature with scales. In this paper, we estimated a heteroskedastic conditional logit and a scale-adjusted latent class model to emphasize three sources of error related to respondent behavior: (1) task sequence, (2) garbage classes, and (3) failing a PC dominance task.

First, heteroskedasticity may occur as individuals' attention span reduces doing tasks consecutively [30, 35]). Interestingly, after controlling for heteroskedasticity, only a few incremental coefficients differ significantly between BWS and PC, which suggests the tasks might be used interchangeably. Second, the members of the garbage class may be indifferent between EQ-5D-5L profiles or respond randomly (i.e., a shuffled deck)[36]. Although these motives are confounded, this class did not express relative attribute importance; therefore, their responses can be disregarded as uninformative. Lastly, respondents who failed the PC dominance task were more likely to belong to a class with a lower scale, which implies that less weight was given to their preference evidence. Overall, the SALC model adjusts the EQ-5D-5L values to better represent the tasks in the middle of the sequence and persons who did not belong to the garbage class or failed the PC dominance test. By controlling heteroskedasticity and heterogeneity, this study produced a Dutch EQ-5D-5L value set on a pits scale that is moderately concordant with the original values. The moderate agreement is in line with our expectation as the study used an online sample with smaller sample size compared to the original study.

This study has several limitations. First, the results of the estimated model were shown on a pits scale rather than on a QALY scale. Second, this study is more of an exploratory study rather than a confirmatory analysis, which complicates the interpretation of p-values or statistical inference more generally. Third, the confounding between taste and scale in choice-based analysis implies that adjusting the scale might not totally control the scale factor from preference coefficients. Also, due to the design with a constant comparator in PC tasks and relatively smaller sample size, our capability to explore heterogeneity in larger dimensions was beyond the scope. Lastly, important variables such as income and time to complete the tasks were missing in the dataset, which would have been good indicators for class membership, as shown in previous studies [18]. Given this, this study is the first attempt to explore heteroskedasticity and heterogeneity in a health valuation study and should aid others considering similar approaches. It is also worth to be mentioned that the SALC model is a certain parametrization of a particular type of disentangling taste and scale. So, the results would be dependent on that particular parametrization and require justified theoretical background.

## Conclusions

In conclusion, this study suggests that proper consideration regarding the sources of variation that affect individuals' decision rules can be included to inform the model analysis in health valuation studies. Considering the demonstrated potential of the case-2 BWS task to produce similar values as of PC, this study produced a Dutch EQ-5D-5L value set on a pits scale that is concordant with the original values by controlling heteroskedasticity and heterogeneity. In order to emphasize the importance of controlling the noises in the dataset, this method should be implemented in future studies with larger sample size and with richer behavioral information.

## Figures and Tables

**Fig. 6:**
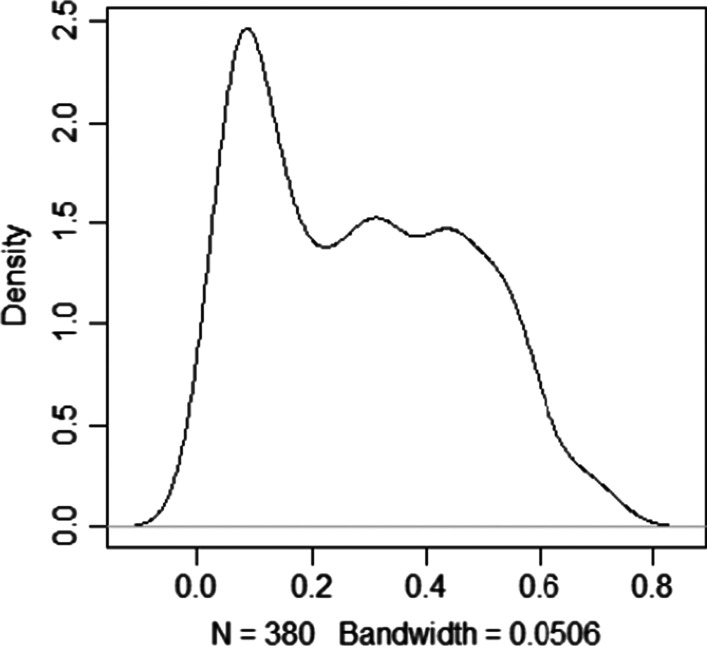
Distribution of Individual grade-of-membership in taste class 1 from 2scale-2taste class SALC model

**Fig. 7:**
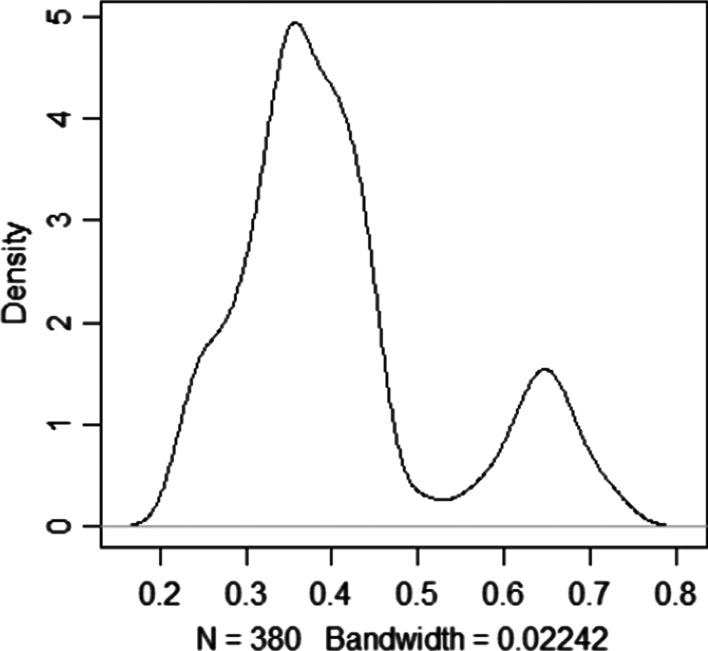
Distribution of Individual grade-of-membership in scale class 1 from 2scale-2taste class SALC model

## Data Availability

The dataset and analysis code are available from the corresponding author upon request.

## Appendix 1

See Figs. 4, 5, 6, 7.

See Tables

**Table 5:** Fraction chosen health states over the comparator state B (24242) and best–worst counts for that health state for each dimension

Health state	DCE	BWS counts				
	Fraction chosen	Mobility	Selfcare	Usual activities	Pain	Depression
(44154)^−,+^	0.211	− 58	− 11	258	− 118	− 71
(51445)^+^	0.253	− 49	270	− 37	− 80	− 104
(33544)^−^	0.271	175	86	− 73	− 113	− 75
(45335)	0.279	− 72	− 7	137	73	− 131
(53352)^−^	0.284	− 82	68	83	− 144	75
(12555)^+^	0.311	263	27	− 61	− 132	− 97
(55214)^+^	0.321	− 94	− 36	51	186	− 107
(54533)	0.332	− 75	− 15	− 70	152	8
(35451)^+^	0.334	89	− 34	− 70	− 139	154
(34225)^−^	0.366	38	− 38	90	75	− 165
(42242)	0.403	− 97	64	117	− 128	44
(15143)^−^	0.442	177	− 40	87	− 200	− 24
(43423)^+^	0.447	− 86	44	− 115	168	− 11
(22434)	0.450	134	114	− 127	8	− 129
(21253)^−^^,+^	0.471	55	213	− 4	− 236	− 28
(25522)	0.516	130	− 53	− 151	67	7
(23115)^−^	0.553	5	14	89	109	− 217
(41511)^−^	0.558	− 117	79	− 121	130	29
(24341)^+^	0.582	61	− 58	12	− 180	165
(52121)^−^	0.605	− 195	22	136	− 29	66
(14412)	0.624	146	− 58	− 174	122	− 36
(32313)^+^	0.700	− 35	6	− 80	212	− 103
(11324)^−^	0.734	182	63	− 44	− 72	− 129
(31132)	0.745	− 52	90	149	− 117	− 70
(13231)	0.811	180	− 60	− 56	− 116	52

^+^Indicates that a health state has only one dimension with a maximum level, ^−^Indicates that a health state has only one dimension with a minimum level

5,

**Table 6:** Full results of the conditional, heteroskedastic model

	Conditional			Heteroskedastic			DCE			BWS			
	Coef	S.E	p-value	Coef	S.E	p-value	Coef	S.E	p-value	Coef	S.E	p-value	p-value
Mobility													
Level 1–2	0.0879	0.0055	< 0.000	0.0726	0.0047	< 0.000	0.0874	0.0035	< 0.000	0.0261	0.0200	0.192	0.001
Level 2–3	0.0331	0.0051	< 0.000	0.0340	0.0046	< 0.000	0.0232	0.0049	< 0.000	0.0478	0.0170	0.002	0.917
Level 3–4	0.1073	0.0056	< 0.000	0.1097	0.0075	< 0.000	0.1103	0.0054	< 0.000	0.1078	0.0192	< 0.000	0.451
Level 4–5	0.0134	0.0055	0.015	0.0059	0.0070	0.398	0.0025	0.0056	0.662	0.0308	0.0181	0.091	0.931
Self-care													
Level 1–2	0.0634	0.0054	< 0.000	0.0623	0.0067	< 0.000	0.0652	0.0037	< 0.000	0.0803	0.0177	< 0.000	0.278
Level 2–3	0.0062	0.0056	0.270	0.0271	0.0053	< 0.000	0.0276	0.0050	< 0.000	0.0545	0.0249	0.028	0.273
Level 3–4	0.0572	0.0054	< 0.000	0.0370	0.0051	< 0.000	0.0498	0.0053	< 0.000	0.0123	0.0236	0.602	0.599
Level 4–5	0.0170	0.0050	< 0.000	0.0164	0.0057	< 0.003	− 0.0002	0.0055	0.974	0.0559	0.0169	0.001	0.999
Usual activity													
Level 1–2	0.0430	0.0052	< 0.000	0.0588	0.0081	< 0.000	0.0685	0.0037	< 0.000	0.0648	0.0235	0.005	0.968
Level 2–3	0.0299	0.0052	< 0.000	0.0280	0.0052	< 0.000	0.0243	0.0051	< 0.000	− 0.0784	0.0237	0.001	0.611
Level 3–4	0.0987	0.0057	< 0.000	0.1002	0.0071	< 0.000	0.1008	0.0055	< 0.000	0.1129	0.0187	< 0.000	0.000
Level 4–5	− 0.0003	0.0055	0.956	− 0.0011	0.0072	0.878	− 0.0046	0.0057	0.423	0.0312	0.0180	0.083	0.999
Pain/discomfort													
Level 1–2	0.0682	0.0053	< 0.000	0.0802	0.0063	< 0.000	0.0854	0.0037	< 0.000	0.0061	0.0177	0.728	0.739
Level 2–3	0.0125	0.0056	0.025	0.0324	0.0074	< 0.000	0.0325	0.0050	< 0.000	0.0637	0.0249	0.010	0.194
Level 3–4	0.1244	0.0055	< 0.000	0.1034	0.0071	< 0.000	0.1160	0.0055	< 0.000	0.0856	0.0221	< 0.000	0.004
Level 4–5	0.0297	0.0051	< 0.000	0.0336	0.0051	< 0.000	0.0139	0.0057	0.015	0.0970	0.0167	< 0.000	0.999
Anxiety/depression													
Level 1–2	0.0649	0.0051	< 0.000	0.0605	0.0047	< 0.000	0.0738	0.0038	< 0.000	0.0106	0.0223	0.636	0.002
Level 2–3	0.0486	0.0051	< 0.000	0.0444	0.0050	< 0.000	0.0319	0.0053	< 0.000	0.0568	0.0166	< 0.000	0.922
Level 3–4	0.0565	0.0055	< 0.000	0.0634	0.0172	< 0.000	0.0623	0.0057	< 0.000	0.0735	0.0193	< 0.000	0.710
Level 4–5	0.0384	0.0055	< 0.000	0.0310	0.0105	0.003	0.0296	0.0057	< 0.000	0.0575	0.0184	0.001	0.926
Ancillary parameters													
SC (Level 0–1)	0.0513	0.0058	< 0.000	0.0349	0.0046	< 0.000							
UA (Level 0–1)	0.0638	0.0058	< 0.000	0.0348	0.0050	< 0.000							
PD (Level0-1)	0.0549	0.0056	< 0.000	0.0207	0.0072	< 0.000							
AD (Level 0–1)	0.0740	0.0056	< 0.000	0.0584	0.0054	< 0.000							
Pits Value	8.7724	0.1138	< 0.000	8.8533	0.3201	< 0.000	8.7669	0.1194	< 0.000	8.5402	0.4977	< 0.000	
Heteroskedasticity*													
Intercept													
Task sequence				0.8402	0.1664	< 0.000							
Task sequence^2				− 0.7439	0.1608	< 0.000							
Task type													
Worst						_							
Best				0.2424	0.0246	< 0.000							
PC				− 0.9931	0.0404	< 0.000							
Log-likelihood	− 32,106.1			− 31,252.3					− 31,160.3				
BIC	64,458.32			62,792.03									
Sample Size	18,620			18,620					18,620				

*Heteroskedastic coefficients presented in log-scale term

6,

**Table 7:** Full result of the SALC model

	Class 1 (Value set)			Class 2 (Garbage class)		
	Coeff	S.E	p-value	Coeff	S.E	p-value
Mobility						
Level 1–2	0.0586	0.0029	< 0.001	0.2954	0.1024	0.004
Level 2–3	0.0290	0.0030	< 0.001	− 0.1132	0.1206	0.348
Level 3–4	0.1205	0.0037	< 0.001	0.1903	0.1351	0.159
Level 4–5	0.0090	0.0038	0.017	− 0.0825	0.1275	0.518
Self-care						
Level 1–2	0.0553	0.0034	< 0.001	− 0.2140	0.1462	0.143
Level 2–3	0.0246	0.0034	< 0.001	0.2202	0.1445	0.127
Level 3–4	0.0630	0.0037	< 0.001	− 0.0745	0.1189	0.531
Level 4–5	0.0082	0.0038	0.030	0.2378	0.1204	0.048
Usual activity						
Level 1–2	0.0516	0.0028	< 0.001	0.0386	0.1477	0.794
Level 2–3	0.0297	0.0031	< 0.001	− 0.0073	0.1628	0.964
Level 3–4	0.1115	0.0037	< 0.001	− 0.0431	0.1458	0.767
Level 4–5	0.0002	0.0037	0.967	0.0544	0.1677	0.745
Pain/discomfort						
Level 1–2	0.0686	0.0030	< 0.001	0.1218	0.1570	0.438
Level 2–3	0.0290	0.0029	< 0.001	− 0.0898	0.1645	0.585
Level 3–4	0.1085	0.0034	< 0.001	0.0930	0.1659	0.575
Level 4–5	0.0346	0.0036	< 0.001	− 0.1657	0.1739	0.341
Anxiety/depression						
Level 1–2	0.0558	0.0028	< 0.001	0.3042	0.1456	0.037
Level 2–3	0.0412	0.0033	< 0.001	0.0648	0.1399	0.643
Level 3–4	0.0753	0.0041	< 0.001	0.0241	0.1664	0.885
Level 4–5	0.0262	0.0039	< 0.001	0.1455	0.1570	0.354
Pits value	6.4267	0.6489	0.000	0.2161	0.0710	0.002
Ancillary parameter						
SC (Level 0–1)	0.0221	0.0027	0.000	0.264	0.148	0.074
UA (Level 0–1)	0.0078	0.0023	0.001	1.039	0.309	0.001
PD (Level 0–1)	− 0.0030	0.0023	0.197	1.540	0.479	0.001
AD (Level 0–1)	0.0248	0.0025	0.000	1.234	0.422	0.003
Heteroskedasticity*	Scale class 1	Scale class 2				
Intercept	1.0067	0.0930	0.123			
Task sequence	1.9589	0.1990	0.220	1.0155	0.3280	0.562
Task sequence^2	− 1.6698	0.2004	0.222	− 0.6295	0.3028	0.519
Task type						
Worst	–	–	–	–	–	–
Best	0.383	0.043	0.045	0.5628	0.0547	0.054
PC	− 1.301	0.047	− 0.049	− 0.8878	0.0955	0.097
Log-likelihood	− 27,969.64					
BIC	56,698.35					
Sample Size	18,620					

*Heteroskedastic coefficients presented in log-scale term

7,

**Table 8:** Full result: Grade of membership (GOM) of the scale-adjusted latent class (SALC)

	GOM for taste class 2 Garbage class (29% of respondents)			GOM for scale class 2 Uncertain class (59% of respondents)		
	Coeff	S.E	p-value	Coeff	S.E	p-value
Intercept	0.5022	0.2092	0.098	0.5453	0.7102	0.117
Female	0.5173	0.1488	0.022	1.0399	0.2529	0.882
Age in years						
16–35	2.4143	0.7634	0.005	1.1669	0.2236	0.554
36–54						
above 55	0.2100	0.1292	0.011	1.4360	0.1712	0.141
Educational attainment						
Low	0.6366	0.3328	0.388	0.8678	0.4312	0.705
Medium						
High	0.9140	0.4058	0.839	0.9635	0.3450	0.911
Chronic Illness						
Yes	1.0643	0.5052	0.895			
VAS score Health						
Below 70	1.8346	0.9661	0.249			
70 >						
Difficulty level						
Failed dominant task				3.1286	0.1814	0.044
Found tasks easy				1.1337	0.5275	0.834
Found tasks hard				0.6152	0.5356	0.140

Results are shown on the odds ratio scale. For education, the lowest group included up to the primary, the medium group included secondary to associates, and the highest group included bachelor’s degrees and above

8.

## Appendix 2

### Scale-adjusted latent class (SALC) model

The scale-adjusted latent class model [20] is an advanced version of the latent class model in the context of stated choice experiment studies. The latent class model assumes that the population can be decomposed into distinct number of latent classes where each class differs by their preferences (different sets of coefficients). The latent class model is restricted by the assumption of constant scale. The SALC model relax the assumption of constant scale and assumes that the population can be decomposed in two overlapping ways: classes defined by S different scales ${\mu }_{s}$ and classes defined by M different effects ${\beta }_{m}$. This would further imply that, despite sharing the same coefficients, within the same effect class, some subjects may display different levels of uncertainty, thereby belonging to different scale classes. So, the probability of choice $j$ by subject $i$ in choice situation $t$, conditional on scale class s and effect class *m* is

$$P(y_{itj} |s,m) = \frac{{e^{{\mu_{s} \beta_{m}^{\prime } x_{itj} }} }}{{\mathop \sum \nolimits_{k = 1}^{{S_{it} }} e^{{\mu_{s} \beta_{m}^{\prime } x_{itk} }} }};j,k \in S_{it}$$

${S}_{it}$ is the choice set that includes objects specific to individual’s choice situation, and for example, in a binary logit model, each ${S}_{it}$ includes two objects. Similar to the latent class model, the SALC model also identifies class memberships in a probabilistic fashion. However, two different sets of covariates have been used to identify effect and scale class membership (${z}_{1i}$ and ${z}_{2i}$, respectively). For the effect class, the class assignment will be associated with individual characteristics that are related to preference. On the other hand, scale classes are differentiated by subject's randomness in behavior (e.g., certainty). So, to identify scale class membership we have considered variables related to subjects’ characteristics that might influence their randomness in behavior. As both sets of covariates includes some common variables, each set includes at least one unique variable to identify the model. As for example, demographics [age, gender, race, ethnicity] and SES[educational attainment, household income]) characteristics influence both preference and scale. However, individual’s current usage of a specific product might influence their preference but not related to any randomness in behavior. On the other hand, behavioral phenomena like time of the day when the survey was completed, and survey completion time period are variables that relate with individual’s randomness in behavior and nothing to do with their taste. In both cases, the covariates are included in multinomial logit models

$$P(m|z_{1i} ) = \frac{{\exp (\delta_{m}^{\prime } z_{1i} )}}{{\mathop \sum \nolimits_{k = 1}^{M} \exp (\delta_{k}^{\prime } z_{1i} )}}$$

$$P(s|z_{2i} ) = \frac{{\exp (\theta_{s}^{\prime } z_{2i} )}}{{\mathop \sum \nolimits_{k = 1}^{S} \exp (\theta_{k}^{\prime } z_{2i} )}}$$

The full choice model of each subject $i$ becomes

$$P\left( {y_{i} |z_{i} ,q_{i} } \right) = \mathop \sum \limits_{s = 1}^{S} P\left( {s|z_{2i} } \right)\mathop \sum \limits_{m = 1}^{M} P\left( {m|z_{1i} } \right) \mathop \prod \limits_{t = 1}^{T} \mathop \prod \limits_{j = 1}^{J} P(y_{itj} |s,m)^{{y_{itj} }}$$

This likelihood function *L* is simply a joint cumulative density function (CDF) made up of choice probabilities, scale class probabilities $P(s|{z}_{i})$, and effect class probabilities $P(m|{z}_{i})$. Hence, the overall log-likelihood function for all subjects becomes

$$\ln L = \mathop \sum \limits_{n = 1}^{N} \ln \left[ {P\left( {y_{i} |z_{i} } \right)} \right] = \mathop \sum \limits_{i = 1}^{N} \ln \left[ {\mathop \sum \limits_{s = 1}^{S} P\left( {s|z_{2i} } \right)\mathop \sum \limits_{m = 1}^{M} P\left( {m|z_{1i} } \right) \mathop \prod \limits_{t = 1}^{T} \mathop \prod \limits_{j = 1}^{J} P(y_{itj} |s,m)^{{y_{itj} }} } \right]$$

The scale—factor can also be modeled by linear equation. The rationale behind this specification is that the scale factor may depend on latent class and/or independent variable. In order to remain the scale parameter as non-negative, we are constraining the scale parameter as $\mathrm{exp}({\mu }_{s})$. So, the scale factor model contains a term for scale class and effect of independent variables (${z}_{3it}$).

$$\mu_{s} = \gamma_{s0} + \mathop \sum \limits_{{z_{3it} = 1}}^{{Kz_{3} }} \gamma_{s1} z_{3it}$$

where for s = 1, the constant term ${\gamma }_{s0}$ is 0 for the identification purpose.

Here in the model, the independent variables are sequence of choice task and time spent per choice task. So, independent variables are task(*t*) specific.

## Abbreviations

BWS: Best–worst scaling
EQ-5D-5L: EuroQol 5 Domains 5 Levels
GOM: Grade-of-membership
HPR: Health preference research
OMEP: Orthogonal main effects plan
PC: Paired comparison
SALC: Scale-adjusted latent class
TTO: Time trade-off

## Footnotes

1 Rotating OMEP coding means permuting the levels of one or more of the attributes such that an equivalent OMEP design is obtained.
